# Double the Trouble - One Infarction After Another: A Case Report of Two Consecutive ST-Segment Elevation Myocardial Infarctions in Two Different Coronary Arteries

**DOI:** 10.7759/cureus.36616

**Published:** 2023-03-24

**Authors:** Zineb Agoumy, Kaoutar Berrag, Aida Soufiani, Nesma Bendagha, Rokya Fellat

**Affiliations:** 1 Cardiology A, Ibn Sina Hospital, Rabat, MAR

**Keywords:** recurrent stemi, coronarography, coronary arteries, culprit lesion, myocardial infarction

## Abstract

In multi-vessel coronary artery disease, concomitant ST-segment elevation myocardial infarction (STEMI) in simultaneous two culprit lesions have been rarely reported. In this regard, the recurrence in a short period of time of a STEMI in a different coronary artery is also rare. We describe the case of a 56-year-old male smoker, who was presented with an anterior STEMI. The coronary angiography demonstrated a significant lesion in the left main coronary (LMC) and an occlusion of the left anterior descending artery (LAD), and was referred for surgery. Four days later, he experienced symptoms of acute ischemia of the inferior territory. A newly formed culprit lesion of the circumflex artery (Cx) was detected and benefited from angioplasty. The patient expired the next day from sudden arrythmia. This case report shows two consecutive STEMI situations in separate coronary arteries, which commonly can occur in atherosclerotic patients with very poor prognosis.

## Introduction

Acute myocardial infarction (AMI) early survivors are at higher risk of developing another ischemic event. Generally, the recurrence of ischemia happens in the case of an unrecognized culprit coronary lesion or an acute stent thrombosis in the event of percutaneous coronary intervention (PCI). However, the occurrence of two STEMI caused by two different culprit lesions within a few days is very rare with few published similar case reports. The accompanying inflammation during an ischemic situation may progress to thrombus formation, which can occur even in an innocent coronary artery.

## Case presentation

**Figure 1:**
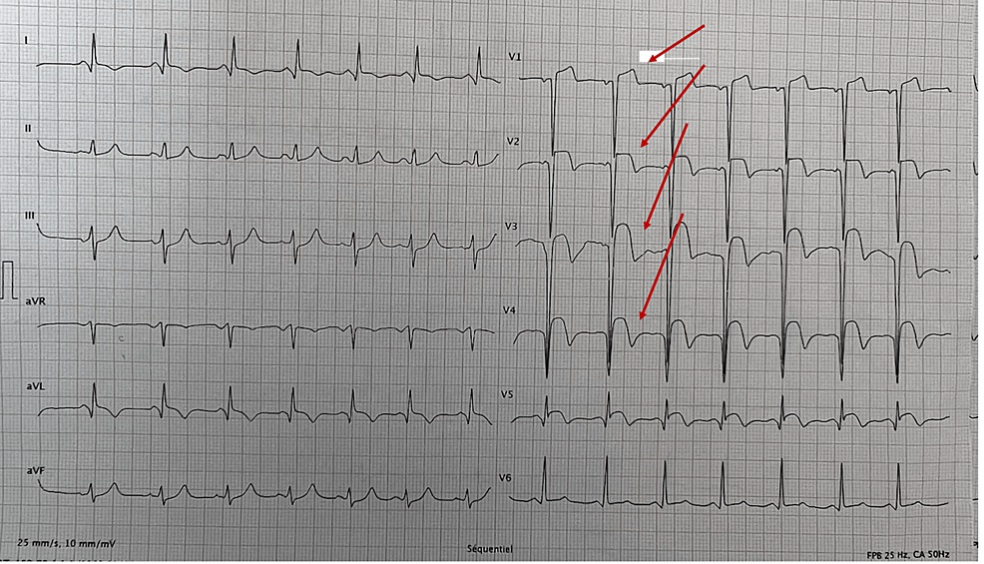
Admission EKG that showing a clear ST-segment elevation in the anterior leads. EKG, electrocardiogram

We present a case of a 56-year-old male, with history of active smoking, who presented with acute chest pain to the emergency department (ED) within hours from its onset. On admission, the patient had a rapid heart rate of 92 bpm with a blood pressure (BP) at 112/80 mmHg. An electrocardiogram (EKG) was immediately performed and showed a significant elevation of the ST segment in the anterior and lateral leads (Figure 1). 

**Figure 2:**
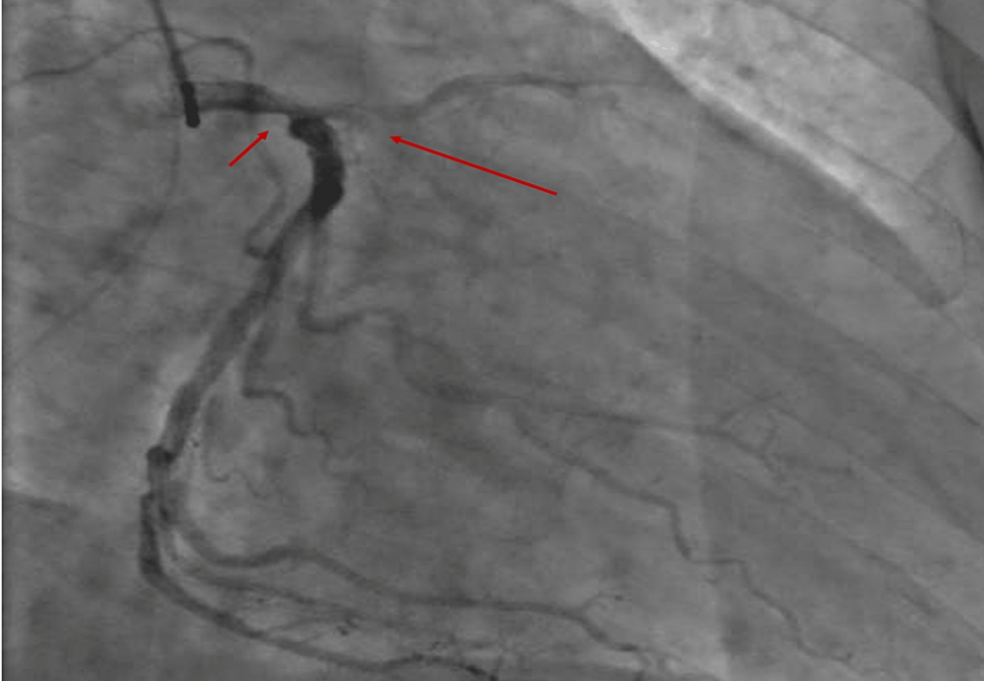
Coronary angiogram on admission in RAO view showing a stenosis of the LMC and a calcified obstruction of the LAD. LMC, left main coronary; LAD, left anterior descending artery

**Figure 3:**
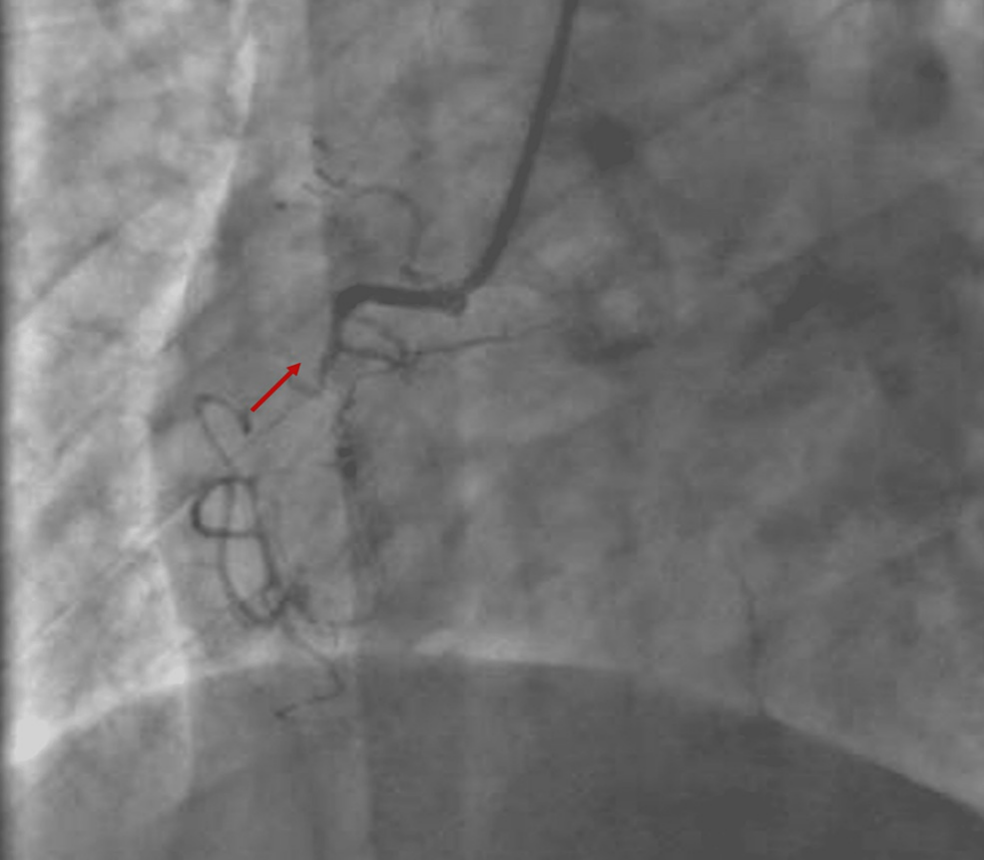
Coronary angiogram on admission in LAO view showing a chronic occlusion of the RCA. RCA, right coronary artery

After initial loading with dual antiplatelet therapy DAPT (clopidogrel and aspirin), the patient underwent emergency coronary angiography. It showed a multivessel disease: 50% stenosis of the LMC right before the bifurcation, calcified lesion in the LAD, and chronic occlusion of the right coronary artery (RCA) (Figures 2-3). Coronary artery bypass grafting (CABG) was recommended five days after ceasing clopidogrel.

**Figure 4:**
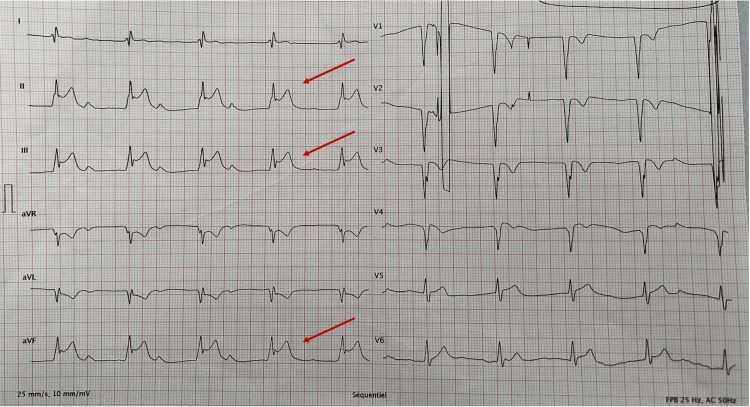
EKG after a few days showing ST segment elevation in the inferior leads. EKG, electrocardiogram

After four days, the patient presented a recurring very intense chest pain with vomiting. His BP dropped significantly with signs of hypoperfusion. Vasopressors were immediately introduced for cardiogenic shock. His EKG revealed a recent ST segment elevation in the inferior leads (Figure 4). 

**Figure 5:**
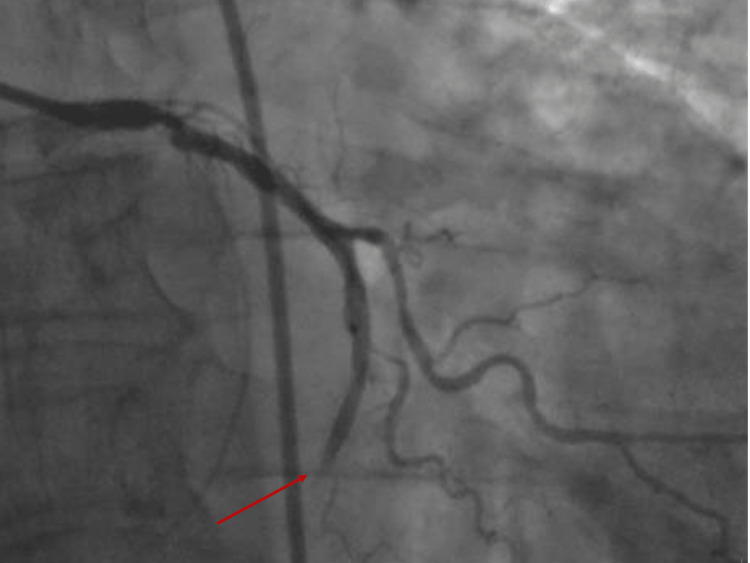
Coronary angiogram is RAO view showing a recent obstructive lesion of the Cx. Cx, circumflex artery

An emergency coronary angiography was conducted and showed a very recent thrombotic lesion in the Cx, considered to be a recent culprit lesion (Figure 5).

**Figure 6:**
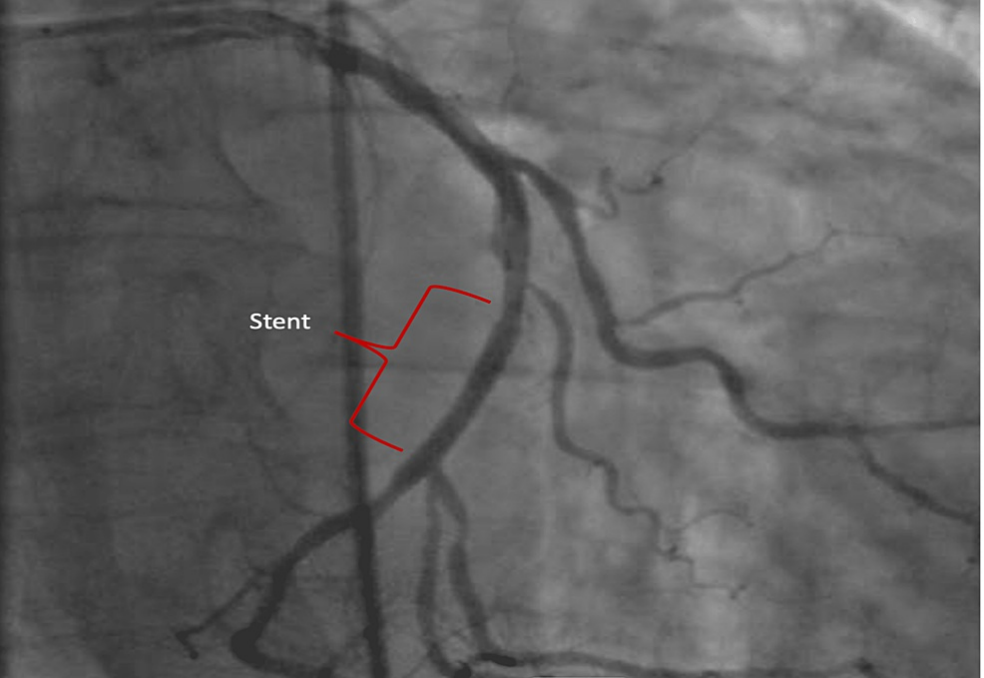
Angiography results after stunting.

Reperfusion was obtained by dilatation and stenting, which restored a TIMI 3 flow (Figure 6).

The patient was then transferred to the intensive care unit (ICU) for surveillance, where progressive weaning from vasoactive drugs was initiated. The follow-up laboratory results showed an important raise in the inflammatory markers: C-reactive protein: 120 mg/L, white blood cells above 18000 elements/mL, correlated to an infectious pulmonary infection. The additional septic shock factor prompted the fast introduction of empiric antibiotics. The immediate clinical evolution was favorable. The infectious markers dropped after a few days of IV antibiotics. However, an early transthoracic echocardiography (TTE) showed a very impaired left ventricular ejection fraction LVEF (35%). The decision was then to keep the patient under DAPT and treat the other coronary lesions with surgery after stabilization. Unfortunately, the patient expired from arrythmia.

## Discussion

The STEMI or acute ST-elevation myocardial infarction occurs when there is an occlusion of the blood flow through the coronary arteries. Ischemia starts as sub-endocardial and progresses to transmural infarct leading to myocardial injury and necrosis. STEMI are commonly reflected in a single coronary artery occlusion after a rupture of an unstable plaque. Nevertheless, there are some uncommon cases of simultaneous STEMIs in two different electric territories depicting two distinct coronary occlusions. It is very rare scenario that can be embedded with high mortality. It is also called deadly double infarction [1]. 

For the same reason, the occurrence of two consecutive myocardial infarctions in two different arteries, where the second culprit lesion is innocent at first, is also a very unlikely scenario. However, a recent study about the characteristics and outcomes of early recurring myocardial infarction (RMI) after AMI admitted that 10% of patients having RMI developed new vessel involvement. Furthermore, patients who were medically managed had a higher chance of developing reinfarction within 90 days than patients who were revascularized [2].

Generally, the recurrence would involve the same electrocardiographic leads, Additionally, most previous descriptions of two recurrent STEMIs are related to acute stent thrombosis [3]. To our knowledge, this is the third case reported of consecutive STEMIs from different culprit lesions in a very short time frame.

Multiple hypothesis may explain this unusual phenomenon, which causes some non-menacing plaques to form a rapid occlusion. It is actually suggested that the persistent inflammatory environment arousing from a myocardial infarction is set to rise the inflammatory markers, leading to disseminated intravascular coagulation (DIC). An additional inappropriate activation of the inflammatory response following AMI caused by auto-immune mechanisms is very likely to maintain the inflammation with rapid progression and predispose further adverse events [4]. The event of a concomitant sepsis in the acute phase is also set to boost the inflammatory response.

As a consequence, medications with anti-inflammatory effects are primordial in preventing reinfarctions. Therefore, an abrupt stoppage of clopidogrel in preparation for surgery is likely to be a perfect window for unfavorable thrombotic progression. Guidelines suggest that patients should discontinue clopidogrel at least five days prior to CABG. Although their early introduction in acute coronary syndrome is primordial to decrease new ischemic events, it also comes with increasing post operative bleeding [5]. 

The alternative in our case, where the patient is considered at high risk of an early fatal event, was to undergo CABG under DAPT. For most patients with chronic occlusions and multivessel disease, bypass grafting beats with clear reductions in death. CABG can be a very feasible and probably safe option, with adequate means of reanimation and prophylactic per-operative platelet transfusion [6].

Moreover, we should consider additional aggravating hemodynamic factors such as hypotension and DIC, which probably result in the decrease of blood perfusion in the coronary arteries. This was the case of a 77-year-old patient, who was found to have a subtotal occlusion of the RCA with 50% stenosis of the LAD and Cx and underwent PCI of the RCA after AMI. The next day, the patient re-suffered and his hemodynamic status became instable. The new coronary angiogram revealed a permeable stent, but a new total occlusion of the LCA. The explanation given by the authors is that complicated sepsis and persistent signs of shock precipitated the progression of the acute thrombosis in the non-culprit lesion. Hence, many theories could be suggested and an eventual study of predictive factors precipitating AMI in a non-culprit lesion during an acute ischemic phase should be considered to help prevent this unfortunate outcome. 

The main takeaway from this case is that patients who present with STEMI should always be considered at high risk of recurrence of an ischemic event in the early days. And the event of transformation of an innocent plaque into a complete thrombotic occlusion is always a dangerous possibility, especially when not treated and in the absence of anti-ischemic medication. We also suggest that patients with AMI requiring CABG should proceed without delay for a clopidogrel-free period with consideration for both ischemic and hemorrhagic risk.

## Conclusions

Having two consecutive episodes of STEMIs in different coronary locations within a few days is an uncommon setting, but comes with high morbidity and mortality. There is still some debate regarding the exact underlying mechanisms. However, it should draw attention to reconsider the treatment strategy in terms of the exact time of management and encourage CABG under clopidogrel therapy in high ischemic risk patients.
